# Diversity and Distribution of Fungal Infections in Rwanda: High Risk and Gaps in Knowledge, Policy, and Interventions

**DOI:** 10.3390/jof10090658

**Published:** 2024-09-18

**Authors:** Claude Mambo Muvunyi, Jean Claude Semuto Ngabonziza, Masaisa Florence, Isabelle Mukagatare, Marc Twagirumukiza, Ayman Ahmed, Emmanuel Edwar Siddig

**Affiliations:** 1 Rwanda Biomedical Centre, Kigali P.O. Box 7162, Rwanda; ayman.ame.ahmed@gmail.com; 2 Department of Clinical Biology, University of Rwanda, Kigali P.O. Box 3900, Rwanda; 3 Research, Innovation and Data Science Division, Rwanda Biomedical Centre, Kigali P.O. Box 7162, Rwanda; 4 Department of Internal Medicine and Hematology, School of Medicine and Pharmacy, College of Medicine and Health Sciences, University of Rwanda, Kigali P.O. Box 7162, Rwanda; 5 Clinical Education and Research Division, Kigali University Teaching Hospital, Kigali P.O. Box 655, Rwanda; 6 Biomedical Services Department, Rwanda Biomedical Centre, Kigali P.O. Box 7162, Rwanda; 7 Faculty of Medicine and Health Sciences, Ghent University, 9000 Ghent, Belgium; 8 Institute of Endemic Diseases, University of Khartoum, Khartoum 11111, Sudan; 9 Unit of Applied Medical Sciences, Faculty of Medical Laboratory Sciences, University of Khartoum, Khartoum 11111, Sudan; emanwelleds389@gmail.com

**Keywords:** fungal infections, human, soil, plants, public health, healthcare, multisectoral one health, global health, food insecurity, Africa

## Abstract

Fungal infections (FIs) are spreading globally, raising a significant public health concern. However, its documentation remains sparse in Africa, particularly in Rwanda. This report provides a comprehensive review of FIs in Rwanda based on a systematic review of reports published between 1972 and 2022. The findings reveal a rich diversity of fungal pathogens, including *Blastomyces*, *Candida*, *Cryptococcus*, *Histoplasma*, *Microsporum*, *Pneumocystis*, *Rhinosporidium*, and *Trichophyton* caused human infections. Candida infections predominantly affect the vagina mucosa, while *Histoplasma duboisi* was linked to disseminated infections. Other pathogens, such as *Blastomyces dermatitidis* and *Rhinosporidium seeberi,* were associated with cerebellar and nasal mucosa infections, respectively. The widespread observation of soilborne fungi affecting bean crops highlights the pathogens’ threat to agricultural productivity, food security, and socioeconomic stability, as well as potential health impacts on humans, animals, and the environment. Of particular importance is that there is no information about FIs among animals in the country. Moreover, the analysis underscores significant limitations in the detection, reporting, and healthcare services related to FIs in the country, indicating gaps in diagnostic capacity and surveillance systems. This is underscored by the predominant use of traditional diagnostic techniques, including culture, cytology, and histopathology in the absence of integrating more sensitive and specific molecular tools in investigating FIs. Developing the diagnostic capacities and national surveillance systems are urgently needed to improve the health of crops, animals, and humans, as well as food security and socioeconomic stability in Rwanda. Also, it is important to indicate severe gaps in the evidence to inform policymaking, guide strategic planning, and improve healthcare and public health services, underscoring the urgent need to build national capacity in fungal diagnosis, surveillance, and research. Raising awareness among the public, scientific community, healthcare providers, and policymakers remains crucial. Furthermore, this report reveals the threats of FIs on public health and food insecurity in Rwanda. A multisectoral one health strategy is essential in research and intervention to determine and reduce the health and safety impacts of fungal pathogens on humans, animals, and the environment.

## 1. Introduction

**Figure 1:**
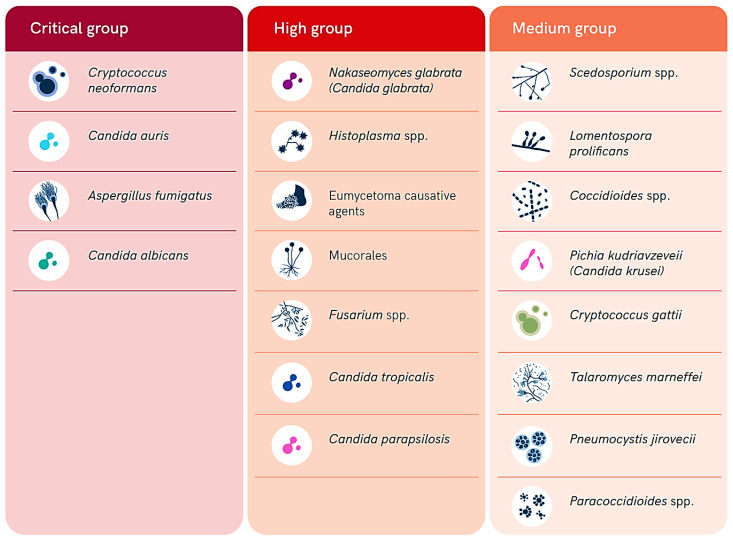
Summary of the WHO list of fungal priority pathogens according to their level of priority (adopted from the WHO open access report [3]).

The fungal population is estimated to include over 3.8 million species distributed throughout the world. However, most of them are understudied and poorly characterized [1]. Most of these species infect humans, animals, and/or plants around the world; nevertheless, they are commonly severely neglected, mainly because of their historically limited distribution in low- and middle-income countries in the tropical and subtropical regions. Fungal infections (FIs) tend to progress gradually, and the delays in detecting them, combined with a lack of effective case management, contribute to the development of their significant burden and impacts, particularly in resource-limited settings. They contribute heavily to diseases morbidity, mortality, and disability, resulting in a severe socioeconomic burden, with over 1.5 million deaths annually attributed to FIs [2]. Therefore, the World Health Organization (WHO) has released a list of fungal pathogens of high priority in healthcare, public health, and research (Figure 1) [3,4,5]. Fungal pathogens in this list are ranked into three categories including critical, high, and medium priority (Figure 1). This ranking was conducted based on the public health significance and emergence of antifungal resistance to the currently available antifungal drugs [3,4,5].

Invasive and noninvasive fungal diseases represent a significant challenge for health systems in the low- and middle-income countries (LMICs), particularly in tropical and subtropical regions of the world. This is of particularly high concern in healthcare settings among the most vulnerable population groups. These groups include immunocompromised individuals, such as those living with human immunodeficiency virus (HIV), hematological malignancies, organ transplant recipients, and patients undergoing long-course immunosuppressive therapy [6,7]. These infections are primarily opportunistic in nature and are characterized by the presence of fungal elements in subcutaneous and deep tissues, as identified through culture or histopathological investigations [8,9].

In low-resource settings like many African countries, the burden of FIs is exacerbated by poor hygiene situations and the high prevalence of HIV, tuberculosis, and poverty, making timely diagnosis and management critical yet challenging in the context of limited budgets allocated to healthcare. The lack of reliable point-of-care tests (POCTs), cost barriers, limited awareness among healthcare providers, delays in diagnosis, and inadequacies in confirmatory blood cultures all contribute to the struggle in addressing invasive fungal diseases (IFDs) effectively [6,7,8,9,10,11]. Early diagnosis and prompt initiation of appropriate antifungal therapy are crucial in combating FIs and reducing associated morbidity and mortality rates [12,13]. However, the scarcity of data on the burden of invasive fungal diseases in Africa hinders efforts to implement targeted interventions and strategies [8]. 

Rwanda, a small landlocked country located in East Africa, is known for its stunning landscapes, including lush green hills, serene lakes, and diverse wildlife. With a population size of over 13 million people, Rwanda is one of the most densely populated countries on the African continent, covering an area of about 26,338 square kilometers [14]. Despite its natural beauty and recent economic development, Rwanda faces various health challenges, including neglected tropical diseases (NTDs) and other infectious diseases [15]. Currently, FIs in Rwanda are receiving no attention, mainly because of lack of evidence to inform policymaking, strategic planning, and the implementation of cost-effective healthcare and public health services. These gaps in knowledge and policy is indicated by the lack of diagnostic or case management guidelines for FIs in the country. 

This literature review aims to provide an overview of the diversity and distribution of FIs affecting humans, animals, and plants in Rwanda. It also summarizes the prevalence, risk factors, and public health implications associated with these infections. By analyzing existing literature and research findings, this review seeks to contribute to fill the current gaps in knowledge and needs for the investment in generating evidence that leads to improvements in policymaking, strategic planning, and the implementation of cost-effective healthcare and public health services in the country and region.

## 2. Materials and Methods

In this research, we followed the updated PRISMA guidelines from 2020 to perform a comprehensive literature review and data mining from databases such as PubMed and Google Scholar, as well as government websites, for grey literature to identify published articles and official reports that contain data about FIs in Rwanda [16,17]. Our search terms included ‘fungal infection and Rwanda’, ‘histoplasmosis and Rwanda’, ‘cryptococcosis and Rwanda’, ‘aspergillosis and Rwanda’, ‘blastomycosis and Rwanda’, ‘pneumocystis pneumonia and Rwanda’, ‘candidiasis and Rwanda’, ‘mucormycosis and Rwanda’, ‘emergomycosis and Rwanda’, ‘talaromycosis and Rwanda’, ‘blastomycosis and Rwanda’, ‘sporotrichosis and Rwanda’, ‘coccidioidomycosis and Rwanda’, ‘fungal keratitis and Rwanda’, ‘allergic fungal rhinosinusitis and Rwanda’, ‘allergic bronchopulmonary aspergillosis and Rwanda’, ‘dermatophytes and Rwanda’, and ‘paracoccidioidomycosis and eumycetoma and Rwanda’. All authors participated in the initial data curation process and screened the publications for relevance. We included retrospective studies, prospective studies, and primarily case series. Case reports were considered for FIs with limited documentation. Furthermore, we conducted ‘snowballing’ by reviewing references in identified papers for additional publications on FIs that might not have been captured in our initial searches. Papers without clear patient origin information or those focusing on FIs beyond Rwanda were excluded. Each case’s data extraction encompassed details such as district, causative agents, infection sites, year, and diagnostic methods.

## 3. Results

Although this systematic review was designed and implemented to investigate the diversity, prevalence, and distribution of FIs that infect any living organism in Rwanda, all reports we found were reporting on FIs among humans and bean crops. 

### 3.1. Burden of Fungal Infection in the Human in Rwanda

**Figure 2:**
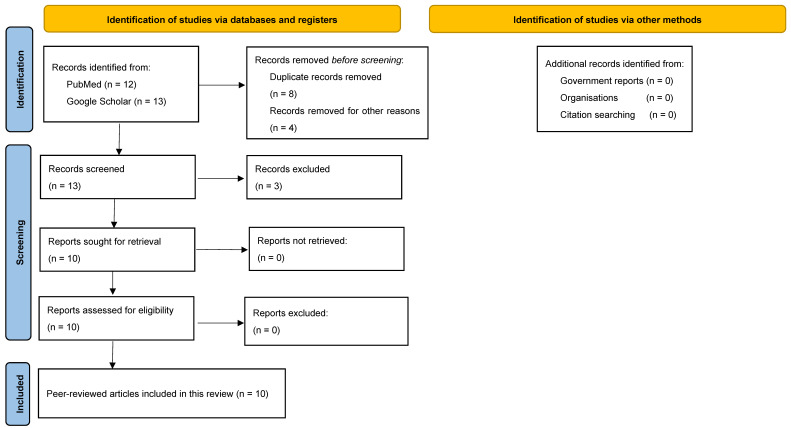
PRISMA flowchart illustrates the process of identification, screening, and inclusion of records that contain information about FIs in Rwanda in this review.

Our systematic search identified 10 reports about FIs in Rwanda that were published between 1972 and 2022 (Figure 2) [18,19,20,21,22,23,24,25,26,27]. Among the identified FIs, there were two reports on candida infections, two reports on *Histoplasma* spp. and dermatophytes, and one report on *Blastomyces dermatitidis*, *Rhinosporidium seeberi*, *Cryptococcus neoformans*, and *Pneumocystis carinii* each.

In terms of infection sites, while *Candida* spp. predominantly affects the vagina, *Histoplasma duboisi* was associated with disseminated infections. On the other hand, *Blastomyces dermatitidis* was linked to cerebellar infections, and *Rhinosporidium seeberi* most commonly affected the nose and conjunctiva. Meanwhile, infections with *Pneumocystis carinii* and *Cryptococcus neoformans* involving the Central nervous system and respiratory system as well as causing dermatophytes affecting the scalp were reported (Table 1).

Candida infections were reported in two districts, specifically Huye and Kicukiro. *Histoplasma duboisi* cases were identified in the Butaro district. *Blastomyces dermatitidis* occurrences were documented in Kigali. Rhinosporidium seeberi incidents were recorded in both Gatsibo and Kirehe districts (Table 1).

When diagnosing FIs, it is important to utilize a combination of traditional and advanced techniques. New technologies such as PCR (polymerase chain reaction) and antigen testing are now widely used for accurate and rapid diagnosis of FIs. This report indicates that the diagnosis of FIs encountered in Rwanda has primarily relied on traditional methods, including culture-based techniques, cytology, histopathology, and immunological testing. Unfortunately, these tools are not accurate enough to identify the fungal species involved in the infection; nonetheless, in most scenarios, effective case management of FIs is species-specific. For *Candida* spp., the diagnosis of infections in Rwanda was mainly performed using culturing techniques and the germ tube method. Furthermore, the identification of *Blastomyces dermatitidis*, *Rhinosporidium seeberi*, and *Cryptococcus neoformans* in Rwanda was based on histopathological techniques, which are not specific tools; therefore, they are not reliable in distinguishing different species within the same genus. Furthermore, *Pneumocystis carinii* is diagnosed using a cytological smear collected from Bronchi-alveolar lavage (BAL) based on the presence of foamy alveolar casts (FACs) and was the distinctive feature that was noted. For dermatophytes, *Trichophyton violaceum*, *Microsporom langeroni*, and *Trichophyton verrucosum* were identified among children, affecting the scalp, and the diagnosis was conducted based on clinical, direct microscopy, and culturing methods (Table 1).

### 3.2. Burden of Fungal Infection in the Plants and Soil in Rwanda

Our search identified only one study focused on soilborne fungi affecting beans in Rwanda, which was conducted by Rusuku and colleagues [27]. This comprehensive survey was carried out across eight prefectures (administrative units) in Rwanda, including Gikongoro, Butare, Gitarama, Kigali, Byumba, Ruhengeri, Gisenyi, and Kibungo, between 1989 and 1990. It revealed a high prevalence and widespread distribution of soilborne fungi pathogenic to common beans, which are the most widely consumed agricultural product in the country (Table 2). Pathogens identified included *Pythium* spp., *Macrophomina phaseolina*, *Rhizoctonia solani*, *Fusarium oxysporum* f. sp. *phaseoli*, and *Sclerotium rolfsii*, with their identification based on various factors such as manifestations, colony characteristics, reproductive structures, and pathogenicity assessments.

A notable percentage of the sampled plants exhibited infection manifestations linked to soilborne fungus across different seasons and regions. This widespread occurrence underscores the significant agricultural impact these fungi could have on common bean production in Rwanda. The presence of these pathogens across multiple prefectures during the study period indicates their widespread threat to crop health and food security. Additionally, it is important to note that certain soilborne fungi, such as *Pythium* spp., *Macrophomina phaseolina*, and *Fusarium oxysporum*, can also cause diseases in humans [28]. These pathogens may pose health risks through various routes, including contamination of food crops or soil exposure.

Thus, understanding the prevalence and distribution of these organisms within agricultural settings is crucial not only for protecting crops but also for safeguarding public health. The findings from this study highlight the need for ongoing monitoring, research, and preventive measures aimed at reducing the impact of soilborne fungi on agricultural productivity and human health [29,30,31].

### 3.3. Underreporting of Fungal Infections in Rwanda

**Figure 3:**
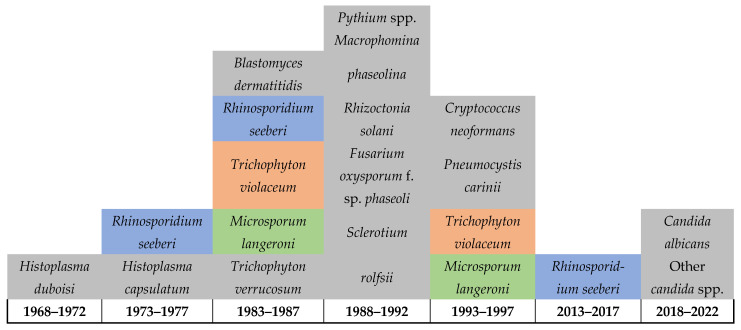
Diagram shows the limited reporting (possibly the detection) of fungal infections in Rwanda up until 2022.

Figure 3 below highlights the severe limitations on reporting FIs in Rwanda, which also suggests limitations in the healthcare services for FIs including the diagnostic capacity, surveillance system, and reporting (Figure 3). This is indicated by the detection of 21 infections in over 50 years throughout the country’s history and is further highlighted by the lack of reporting on any FIs among humans, animals, or in the environment between 1997 and 2013. Of particular note is that daily practices in the country, such as that of most of poor communities in Rwanda, include using motorbikes for their daily transportation, which requires extensive reuse of helmets, as this is required for riders’ safety. Such practices are expected to facilitate the sharing of skin-related pathogens and intensifies the transmission of dermal infections including fungal, bacterial, parasitic, and viral. Therefore, there is an urgent need for improving the diagnostic services for FIs including the integration of molecular and genomic techniques, implementing a national surveillance to monitor the dynamics of these pathogens and the risk factors associated with their transmission in the country. This will help in developing evidence-based decisions and policymaking as well as strategic planning and resource mobilization to improve the health of affected communities.

**Figure 4:**
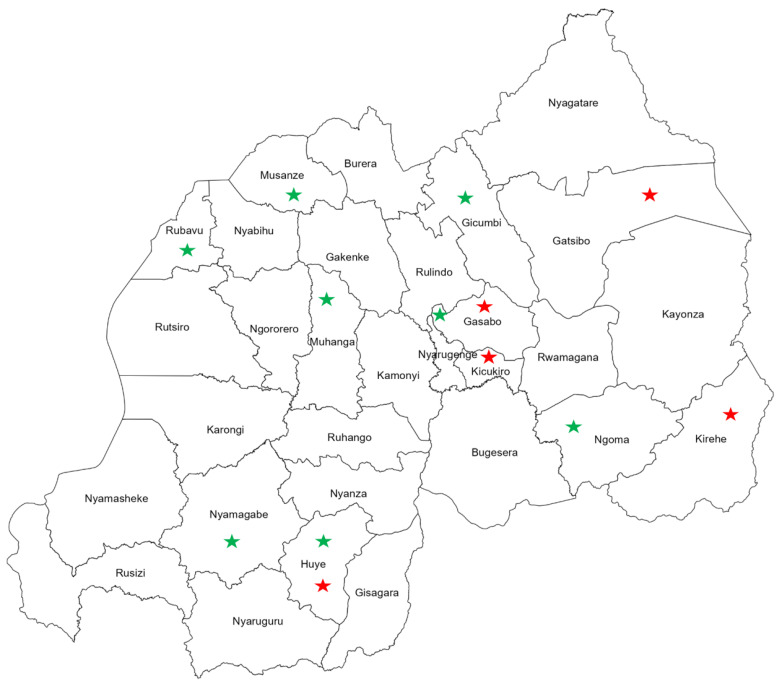
Map of Rwanda shows the geographical distribution of fungal infections among humans, indicated with red stars, and beans (crop), indicated with green stars.

Interestingly, visualizing the geographical distribution of FIs reported from Rwanda revealed countrywide spread of fungal pathogens with distinct spatial clustering of FIs among the populations of human and beans (crop). Human infections with fungal pathogens were mainly reported from the eastern and central provinces of the country (Figure 4). Meanwhile, FIs among agricultural plants and products were distributed in the southern and northern provinces throughout the western region (Figure 4).

## 4. Discussion

The information provided in this review highlights the significant burden of FIs in Rwanda, encompassing various types of fungal pathogens affecting both humans and agricultural crops [18,19,20,21,22,23,24,25,26,27]. The reports indicate the presence of FIs caused by *Candida* spp., *Histoplasma duboisi*, *Blastomyces dermatitidis*, *Rhinosporidium seeberi*, *Cryptococcus neoformans*, *Pneumocystis carinii*, and dermatophytes in different regions of Rwanda. It is clear that diagnosing these FIs requires a mix of traditional and advanced techniques, with some reliance on classical methods like culture-based diagnostics, cytology, histopathology, and immunological testing.

In addition to the human health implications, a study on soilborne FIs in Rwanda reported on the prevalence of pathogens, such as *Pythium* spp., *Macrophomina phaseolina*, *Rhizoctonia solani*, *Fusarium oxysporum* f. sp. *phaseoli*, and *Sclerotium rolfsii*. These pathogens were reported to mainly affect beans, the main food item of the Rwandan diet. This indicates the serious threat of FIs to food security in the country. The widespread distribution of these pathogens across various regions and seasons poses a significant threat to common bean production, emphasizing the need for continuous monitoring, research, and preventive measures [27].

Despite the widespread distribution of several FIs like aspergillosis, Candida peritonitis, fungal keratitis, *fungal tracheobronchitis*, trichosporonosis, and eumycetoma in the region, so far, none of these fungal pathogens or diseases were reported in Rwanda. This suggests severe gaps in knowledge and evidence about the presence, distribution, and health and socioeconomic impacts of FIs in the country, urging for the urgent need of building the national capacity in the diagnosis, surveillance, research, and case management as well as public health interventions for the prevention and control of FIs in Rwanda. Considering that FIs affect humans, animals, and environmental health as well as food security and socioeconomic stability, cost-effective health policies, strategic planning, and the implementation of interventions should be implemented through a multisectoral one health strategy [31], especially considering that, due to several risk factors including change in climate, living environment, and land use and land cover, infectious diseases are increasingly emerging and spreading throughout the country, underscoring the need for implementing a one health strategy [32,33,34,35,36,37].

Interestingly, the impact of fungal diseases on morbidity and mortality in Africa, exacerbated by weak health systems, adds another layer of complexity to the challenges faced in combating these infections in Rwanda. The World Health Organization’s publication of the fungal priority pathogens list underscores the importance of research, development, and public health action in addressing FIs [3,4,5]. The prioritization of common fungal pathogens in Africa as critical and high priority highlights the urgency of the situation, emphasizing the need for enhanced research, diagnostic capabilities, and treatment strategies within Rwanda and other African countries [3]. By aligning with the WHO’s guidance and focusing on these priority pathogens, Rwanda can strengthen its efforts in managing and controlling FIs, ultimately improving healthcare outcomes and agricultural sustainability.

Furthermore, laboratory diagnosis of FIs in Africa presents a significant challenge due to limited resources, access to essential diagnostic tools, and lack of necessary expertise [38,39]. A combination of tests, including microscopy, culture, serology, antigen tests, molecular tests, and histopathology, is typically utilized. Imaging techniques such as X-rays, ultrasound, MRI, and CT scans play a crucial role in diagnosing invasive and chronic fungal diseases, although they may not be reliable for certain conditions like allergic fungal diseases. The diagnostic methods that were used in the detection of FIs in Rwanda were predominantly culturing, microscopy, or histopathology. Though microscopy offers a quick turnaround time, it suffers from low sensitivity, requiring a high level of expertise for accurate interpretation. On the other hand, culturing is widely regarded as the gold standard for fungal diagnosis, yet it is time-consuming, prone to contamination, and some species may not grow in standard blood culture conditions. Therefore, more investment should made be into incorporating the use of molecular tools and genomic analysis for the early detection and monitoring of drug sensitivity among FIs [40]. This issue is further intensified by changes in the clinical manifestations of diseases and co-infections that alter or mask the original cardinal symptoms that are essential for making accurate differential diagnoses [41]. This necessitates improving the diagnostic capacity by integrating diagnostic tools with high sensitivity and specificity to differentiate and characterize infections and co-infections with various pathogens.

Moreover, conducting fungal cultures poses challenges in low-income countries due to inadequate maintenance and upkeep of laboratory facilities and equipment, leading to frequent environmental contamination. The limited access to commercially selected media further complicates the process, hindering accurate identification and characterization of fungal species. Fungal culturing emerged as the most frequently conducted laboratory diagnostic assay in a majority of African countries.

In resource-limited settings like Africa, the availability of diagnostic services is sparse, with fewer than 10 African countries having national surveillance programs for FIs and even fewer possessing reference diagnostic mycology laboratories. The disparity in diagnostic capabilities across the continent is evident, with many diagnostic tests designed for high-income countries and not readily accessible in Africa. While MRI and CT scans are costly, X-rays, a more affordable option, remain unavailable in many primary health centers, placing a financial burden on patients for diagnosis [42]. A recent survey across 50 African countries highlighted variations in diagnostic practices, showing higher rates of chest X-rays and CT scans in the public sector compared to the private sector [43].

## 5. The Need for a Regional Center of Excellence in Research and Development to Improve the Early Detection, Surveillance, and Case Management of Fungal Infections in Africa

Assessing the diagnostic capacity for FIs in Africa in collaboration with Africa CDC, the Global Action for Fungal Infections (GAFFI), and other stakeholders that was launched in Rwanda during the 2nd International Conference on Public Health in Africa (CPHIA), in 2022, has urged for strengthening the regional capacity [44]. It highlighted the need for investment in strengthening the diagnostic capacity, surveillance, policymaking, and improving the case management as well as implementation of preventive and control measures to reduce the transmission of FIs. Additionally, the survey conducted by the European Confederation of Medical Mycology (ECMM) and the International Society for Human and Animal Mycology (ISHAM) revealed that only a small percentage of institutions in Africa meet the minimum laboratory standards for clinical mycology [7]. These constraints highlight the urgent need for improved infrastructure, training, and access to essential resources in low-resource settings to enhance the accuracy and efficiency of fungal diagnostics [7]. However, to create cost-effective improvement and capacity building, there is an urgent need for establishing a regional center of excellence that can lead to building the diagnostic capacity in the region. This capacity-building plan should include training healthcare providers and diagnostic supervisors and technicians in aspects related to the clinical manifestations of FIs, their differential diagnosis, and a cost-effective diagnostic algorithm. Although such comprehensive and high quality training is not currently available in Africa, in order to ensure developing competency in locally prevalent FIs and retaining trained professionals, it is important to implement such training in the region rather than abroad [45]. Furthermore, this center would serve as a reference diagnostic laboratory to support all countries through a well-established coordination mechanism that could be facilitated by establishing a network of laboratories that provide diagnostic and research services in the region [46]. Moreover, this center would enhance the implementation research in the region with a focus on FIs to generate evidence to inform decision-making, strategic planning, and resource mobilization and guide interventions as well as foster research and development of novel prevention, control, and treatment modalities.

To fully investigate the fungal diversity, distribution, and impacts on humans, animals, and environmental health, there is a need for systematic and comprehensive investigations [31,47,48,49]. This will require the deployment of advanced, robust diagnostic technology, such as whole genome sequencing combined with environmental DNA sampling to detect even the residual DNA of the different species of Fungi in the area [50,51,52,53,54]. Moreover, considering the devastating socioeconomic impacts of severe FIs, including amputation-induced disability and food insecurity due to extreme infestations of FIs on agriculture products, there is a crucial need to investigate the socioeconomic burden of FIs on patients and health systems as well as the development of effective mitigation and rehabilitation mechanisms [55,56,57,58,59].

Additionally, considering that there is little evidence about the social awareness and knowledge among healthcare providers and public health leaders about the diversity, distribution, risk, and personal protection measures to prevent and control FIs in Africa, it is important to invest in social and qualitative research to investigate and improve the knowledge, attitude, and practices among the different demographic structures and groups at risk [60,61].

Therefore, it is envisioned that this center will support the health systems in generating evidence from the field that are crucially needed to develop evidence-based health policies, inform strategic planning and resource mobilization, and guide the implementation of effective interventions. This will help reduce the burden of FIs in Africa. More importantly, due to the wide range of hosts for FIs including humans, animals, plants, and agricultural products, which particularly impact health and food security, the investment on improving the surveillance, prevention, and control of FIs should be a top multisectoral priority, especially considering that FIs could impede countries from achieving the sustainable development goals (SDGs), particularly the goals directly related to health, food security, and socioeconomic stability including no poverty, zero hunger, good health and well-being, decent work and economic growth, reduced inequality, sustainable cities and communities, responsible consumption and production, climate action, and life on land [62,63,64,65].

## 6. Conclusions

This review underscores the significant burden and wide geographical spread of FIs in Rwanda, highlighting the diversity of fungal pathogens threatening the health of both human and agricultural products as well as food security and socioeconomic stability in the country. The findings reveal critical gaps in up-to-date knowledge and limitations of the diagnostic capacity and surveillance systems, hindering the overall understanding of FIs in Rwanda. This, in turn, creates challenges for the development of informed policymaking and strategic planning as well as the implementation of cost-effective healthcare and public health services. While traditional diagnostic techniques, including culturing and histopathology, might be useful in some settings, there is an urgent need to integrate advanced molecular and genomic techniques for enhancing early detection, monitoring the dynamics of FIs across different host species, and preparing for and preventing the emergence and local establishment of invasive fungal pathogens. The widespread distribution of soilborne fungi poses additional challenges, threatening the agricultural landscape and, consequently, the nutrition, food security, and livelihood of the Rwandan population and increases health disparity and inequality, because poor communities and individuals involved in farming are at a high risk. Moreover, the current lack of surveillance, healthcare, and public health services for FIs in Rwanda threaten the country’s progress toward achieving the sustainable developmental goals (SDGs). Urgent investment is needed to improve the surveillance, prevention, and control of FIs in Rwanda and help improve human, animal, and environmental health, food security, and socioeconomic growth in the country. 

## Figures and Tables

**Table 1:** Summary of the diversity, geographical distribution, year of report, and site of isolation of fungal infections reported in humans in Rwanda between 1972 and 2022.

Reference	Fungal Name	Site of Isolations	Year	Diagnostic Tool Used	District
Chrysostome et al. [19]	*Candida albicans* Other *candida* spp.	Vaginal mucosa	2021–2022	Culture technique	Kicukiro District
Ndoricyimpaye et al. [18]	*Candida albicans* Other *candida* spp.	Vaginal mucosa	2020	Culture technique	Huye District
Izimukwiye et al. [23]	*Rhinosporidium seeberi*	Nasal mucosa	2016	Histopathology	Kirehe District
Izimukwiye et al. [23]	*Rhinosporidium seeberi*	Nasal mucosa	2014–2015	Histopathology	Gatsibo District
Batungwanayo et al. [24]	*Cryptococcus neoformans*	RLung (Lower respiratory tract) and Brain	1994	Culture and histopathology	Kigali District
Batungwanayo et al. [24]	*Pneumocystis carinii*	RLung (lower respiratory tract)	1994	Cytology	Kigali District
Bugingo. [26]	*Trichophyton violaceum*	Scalp	1993	Direct microscope and culture	Butare District
Bugingo. [26]	*Microsporum langeroni*	Scalp	1993	Direct microscope and culture	Butare District
Raftopoulos et al. [22]	*Blastomyces dermatitidis*	Cerebellar	1986	Immunology, Histopathology	Kigali District
Izimukwiye et al. [23]	*Rhinosporidium seeberi*	Conjunctiva	1986	Histopathology	NA
Buginco. [25]	*Trichophyton violaceum*	Scalp	1983	Direct microscopic	Butare District
Buginco. [25]	*Microsporum langeroni*	Scalp	1983	Direct microscopic	Butare District
Buginco. [25]	*Trichophyton verrucosum*	Scalp	1983	Direct microscopic	Butare District
Izimukwiye et al. [23]	*Rhinosporidium seeberi*	Nasal	1975–1977	Histopathology	NA
Dierckxsens et al. [21]	*Histoplasma capsulatum*	NA	1976	NA	NA
Jadin et al. [20]	*Histoplasma duboisi*	Disseminated infection	1972	Culture technique	Butaro District

**Table 2:** Summarizes the diversity, geographical distribution, year of report, and site of isolation of fungal infections reported in beans in Rwanda between 1972 and 2022.

Reference	Fungal Name	Site of Isolations	Year	Diagnostic Tool Used	District
Rusuku et al. [27]	*Pythium* spp.	Bean	1989–1990	Manifestations, colony characteristics, reproductive structures, and pathogenicity assessments	Nyamagabe, Butare, Muhanga, Kigali, Byumba, Ruhengeri, Gisenyi, and Kibungo
Rusuku et al. [27]	*Macrophomina* *phaseolina*	Bean	1989–1990	Manifestations, colony characteristics, reproductive structures, and pathogenicity assessments	Gikongoro, Butare, Gitarama, Kigali, Byumba; Ruhengeri, Gisenyi, and Kibungo
Rusuku et al. [27]	*Rhizoctonia solani*	Bean	1989–1990	Manifestations, colony characteristics, reproductive structures, and pathogenicity assessments	Gikongoro, Butare, Gitarama, Kigali, Byumba; Ruhengeri, Gisenyi, and Kibungo
Rusuku et al. [27]	*Fusarium oxysporum* f. sp. *phaseoli*	Bean	1989–1990	Manifestations, colony characteristics, reproductive structures, and pathogenicity assessments	Gikongoro, Butare, Gitarama, Kigali, Byumba, Ruhengeri, Gisenyi, and Kibungo
Rusuku et al. [27]	*Sclerotium* *rolfsii*	Bean	1989–1990	Manifestations, colony characteristics, reproductive structures, and pathogenicity assessments	Gikongoro, Butare, Gitarama, Kigali, Byumba, Ruhengeri, Gisenyi, and Kibungo

## Data Availability

All data used for the development of this review is available online.
